# Garadacimab and the future of hereditary angioedema prophylaxis: a step upstream in the bradykinin pathway

**DOI:** 10.1097/MS9.0000000000005029

**Published:** 2026-06-04

**Authors:** Harshika Khaim Chandani, Syeda Fadak Zahra Hujjat, Devya Khaim Chandani, Muhammad Saad Khan, Abubakr Mahmoud

**Affiliations:** a Department of Medicine, Jinnah Sindh Medical University, Karachi, Pakistan; b Department of Medicine, Allama Iqbal Medical College, Lahore, Pakistan; c Department of Medicine, Shaheed Mohtarma Benazir Bhutto Medical College (SMBBMC), Karachi, Pakistan; d Department of Medicine, University of Khartoum, Khartoum State, Sudan

**Keywords:** bradykinin, FXIIa inhibitor, garadacimab, monoclonal antibody, prophylaxis

## Abstract

Hereditary angioedema (HAE) is a rare autosomal dominant disorder characterized by recurrent, potentially life-threatening episodes of bradykinin-mediated angioedema affecting the skin, gastrointestinal tract, and upper airway. Despite the availability of several prophylactic therapies, there is a need for effective and convenient treatment options. On 21 June 2024, the U.S. Food and Drug Administration approved Andembry™ (garadacimab-gxii), a first-in-class monoclonal antibody that inhibits activated Factor XII (FXIIa), for prophylaxis of HAE attacks in patients aged ≥12 years. By targeting FXIIa, garadacimab acts upstream in the kallikrein–kinin pathway, suppressing bradykinin generation and preventing vascular permeability associated with angioedema. Clinical trials demonstrated substantial reductions in attack frequency, with the phase 3 VANGUARD study reporting a 91.5% reduction in HAE attacks and that approximately 77% of patients remain attack-free during the study period. Garadacimab is administered as a 200-mg subcutaneous injection once monthly and has a favorable pharmacokinetic profile with an elimination half-life of approximately 18–20 days. Adverse events were generally mild, including headache, nasopharyngitis, and injection-site reactions. The approval of garadacimab represents an important advancement in HAE prophylaxis, offering a novel upstream mechanism, convenient dosing, and potential improvement in quality of life of the patients.

## FDA approves garadacimab: a novel prophylactic therapy for hereditary angioedema

Andembry™ (garadacimab-gxii) was approved by the U.S. Food and Drug Administration (FDA) on *21 June 2024*, to prevent hereditary angioedema (HAE) attacks in patients 12 years of age and older. Developed by *CSL Behring*, garadacimab is the first approved therapy to inhibit *activated Factor XII (FXIIa)*, an upstream component of the kallikrein–kinin cascade, thus representing a paradigm shift in the prophylaxis of HAE^[^1^]^. This manuscript has been developed in accordance with the TITAN Guidelines (2025)^[^2^]^. Relevant literature on hereditary angioedema and garadacimab was identified through search in PubMed and Google Scholar using keywords such as “hereditary angioedema,” “garadacimab,” and “factor XIIa inhibitor.” Priority was given to peer-reviewed clinical trials, major review articles, and regulatory reports describing the efficacy, safety, and pharmacological profile of garadacimab.

## Understanding hereditary angioedema: a rare bradykinin-mediated disorder

Hereditary angioedema (HAE) is a rare autosomal dominant disorder characterized by recurrent, unpredictable episodes of localized swelling (angioedema) affecting the skin, gastrointestinal tract, upper airway, and, less commonly, the urogenital tract. Unlike allergic angioedema, HAE is non-pruritic, non-pitting, and typically unresponsive to antihistamines, corticosteroids, or epinephrine, owing to its distinct underlying mechanism that is excessive bradykinin production. The global prevalence is estimated at 1 in 50 000 people, though it may be underdiagnosed due to clinical overlap with other forms of angioedema^[^3^]^. There are three main types of HAE: Type I HAE, caused by low levels of functional C1 esterase inhibitor (C1-INH); Type II HAE, where C1-INH levels are normal but functionally defective; HAE with normal C1-INH, which may involve mutations in genes such as F12, PLG, KNG1, ANGPT1, or MYOF, and is more common in females and may be estrogen-sensitive^[^4^]^. C1-Deficiency or dysfunction of C1-INH leads to unregulated activation of Factor XII, which converts prekallikrein to kallikrein. Kallikrein, in turn, cleaves high molecular weight kininogen (HMWK) to produce bradykinin, a potent vasodilator that increases vascular permeability, resulting in angioedema. Common clinical manifestations include facial swelling (eyelids, lips, and tongue), gastrointestinal pain (colicky cramps, nausea and vomiting), and laryngeal edema which is a medical emergency that can cause fatal airway obstruction. Physical trauma (such as dental treatments), mental stress, infections, menstruation, and specific drugs like ACE inhibitors or estrogens are examples of triggers^[^5^]^.

## Diagnosis and psychological impact

HAE has a substantial socioeconomic and psychological cost. People who suffer from anticipatory anxiety worry that they might have a laryngeal attack at any moment. Studies show impaired health-related quality of life, increased school/work absenteeism, and high use of emergency care^[^6^]^. Unlike urticaria-related angioedema, HAE does not involve wheals or itching, and patients often report a prodromal phase with symptoms such as fatigue or tingling. Misdiagnosis is common. Diagnosis is confirmed by measuring C4 levels (typically low during and between attacks), C1-INH concentration, and C1-INH function. Genetic testing can aid in ambiguous or normal C1-INH cases^[^7^]^.

## Mechanism of action and pharmacokinetics

The contact activation system plays a central role in the pathophysiology of HAE. Bradykinin is released from HMWK by the serine protease FXIIa, which also activates prekallikrein to kallikrein. Bradykinin causes edema by binding to endothelial cells’ B2 receptors, which increases vascular permeability. Garadacimab functions upstream by blocking FXIIa, which suppresses the entire bradykinin-generating cascade at its source, in contrast to other preventive medications such lanadelumab (a kallikrein inhibitor) and Cinryze/Haegarda (a C1-INH substitute)^[^4^]^. This upstream inhibition could result in increased efficacy and more widespread attack prevention. Following subcutaneous dosing, garadacimab exhibits a bioavailability of approximately 49.7%, with peak plasma levels occurring approximately 7 days following the dose. It exhibits delayed systemic clearance, a lengthy elimination half-life of 18–20 days, and dose-proportional pharmacokinetics. Every population has the same pharmacokinetic profile^[^8^]^.

## Dosing and administration

Once a month, *a set dose of 200 mg* of garadacimab is given subcutaneously. Its extended half-life (18–20 days) guarantees ongoing suppression of FXIIa, enabling reliable preventative protection^[^9^]^. Self-administration empowers patients and encourages adherence, which is especially beneficial for working adults and teenagers.

## Clinical evidence, efficacy, safety, and enhancing quality of life

Three subcutaneous dosages of garadacimab (75, 200, and 600 mg) given to patients with HAE every 4 weeks (after an IV loading dose) were evaluated in a double-blind, placebo-controlled research. With a good safety record and no severe side effects, all dosage groups demonstrated notable drops in monthly attack rates when compared to placebo^[^10^]^. An open-label extension lasting more than 44 weeks beyond the initial Phase 2 trial assessed the long-term safety and effectiveness of patients staying on a monthly dosage of 200 or 600 mg of garadacimab. The safety configuration stayed constant, attack rates stayed close to zero, and increases in health-related quality of life (HRQoL) were maintained^[^11^]^. In HAE patients with a history of at least two attacks per month, the safety and effectiveness of 200 mg garadacimab monthly dosage versus placebo were assessed in the pivotal Phase 3 VANGUARD study. Over the course of 6 months, 77% of subjects were entirely attack-free. The study demonstrated a 91.5% decrease in attack frequency^[^12^]^. Significant improvements were also seen in secondary outcomes, including length, severity, and usage of rescue medication.

The majority of adverse events were mild to moderate and included headache, nasopharyngitis, and injection site responses. Crucially, neither anaphylactic nor thrombotic events were noted^[^13^]^, confirming garadacimab’s good short-term safety profile. Patients frequently suffer from social disengagement, anticipatory worry, and interruptions in their work or schooling.

## Comparative perspective: how does it stand?

Garadacimab enters a competitive landscape with several prophylactic agents like Lanadelumab (Takhzyro) is a monoclonal antibody against kallikrein, berotralstat (Orladeyo) is an oral kallikrein inhibitor, and C1-INH products (Cinryze, Haegarda) replace the deficient inhibitor. Key characteristics of currently available prophylactic therapies for hereditary angioedema are summarized in Table 1. While all reduce attack frequency, garadacimab offers a mechanistic innovation with potentially broader blockade of the contact pathway. Head-to-head trials are not yet available, but indirect comparisons suggest similar or better efficacy with benefit of monthly dosing^[^14^]^.

**Table 1:** Key differences between currently available prophylactic therapies for HAE.

Drug	Mechanism	Route	Dosing	Key advantage
Garadacimab	FXIIa inhibitor	Subcutaneous	Monthly	Upstream pathway inhibition
Lanadelumab	Kallikrein inhibitor	Subcutaneous	Every 2–4 weeks	Proven long-term data
Berotralstat	Kallikrein inhibitor	Oral	Daily	Oral therapy
Cinryze/Haegarda	C1-INH replacemnt	IV/SC	Every 3–4 days	Direct replacement

## Limitations and future considerations

Although garadacimab demonstrates remarkable efficacy in reducing HAE attacks, several questions remain. The long-term inhibition of FXIIa may theoretically influence coagulation pathways and extended safety surveillance will be essential. In addition, the cost and accessibility of biologic therapies remain major barriers to global implementation. Despite its innovation, certain concerns remain including long-term safety of FXIIa inhibition needing evaluation, particularly regarding potential effects on thrombosis and wound healing; cost and access being prohibitive, particularly in low-resource settings; and real-world data on diverse populations, pregnancy, and pediatric use below 12 years of age being lacking. Furthermore, in remote or underdeveloped locations, biologic treatments may be difficult to get and need cold-chain storage^[^11^]^. Beyond HAE, garadacimab’s ability to modulate FXIIa opens up new prospects for illnesses driven by the contact system. It is being studied for specific types of *inflammatory thrombosis, COVID-19-associated acute respiratory distress syndrome (ARDS)*, and *idiopathic non-histaminergic angioedema*^[^8,14^]^.

## Conclusion

The treatment of hereditary angioedema has advanced significantly with the FDA’s approval of AndembryTM (garadacimab-gxii). It may enhance patient satisfaction and illness control due to its unique upstream mechanism, potent clinical efficacy, and easy dosing schedule. Garadacimab may represent an important addition to current prophylactic therapies. Garadacimab is a medication that rethinks the way we approach HAE prophylaxis by switching from reactive disease control to proactive molecular interception. Future research could broaden the therapeutic applications of garadacimab to include other illnesses driven by the bradykinin and contact systems. However, accessibility must keep pace with innovation. Biologics’ exorbitant price, cold-chain constraints, and unequal worldwide distribution may restrict patient access, especially in low- and middle-income nations. To guarantee fair access, future plans should give priority to global supply chain resilience, patient aid initiatives, and pricing methods. Further studies and broader accessibility may determine its role in future treatment guidelines. This would change the field of HAE prophylaxis and may have an impact on how other uncommon immunovascular illnesses are treated.

## Data Availability

No data were generated for this manuscript.

## Research registration unique identifying number (UIN)

Not available.

## References

[1] US Food and Drug Administration. FDA approves Andembry (garadacimab-gxii) for hereditary angioedema. Silver Spring (MD): FDA; Accessed 21 June 2024. https://www.fda.gov

[2] TITAN Group. Agha RA Mathew G Rashid R. Transparency in the reporting of Artificial INtelligence – the TITAN guideline. Prem J Sci 2025;10:100082.

[3] Santacroce R D’Andrea G Maffione AB. The genetics of hereditary angioedema: a review. J Clin Med 2021;10:2023. 34065094 10.3390/jcm10092023 PMC8125999

[4] Smith D Nguyen R. SERPING1 mutations and HAE-nC1-INH variants: a genetics overview. PLoS Genet 2023;19:e1010384.

[5] Thompson AR Davis C. Clinical features and mortality risk of laryngeal edema in HAE. JAMA Intern Med 2022;182:245–51.35099499

[6] Banerji A Craig T Riedl M. Long-term disease burden in hereditary angioedema: findings from the VANGUARD study. Allergy Asthma Proc 2023;44:23–30.

[7] Maurer M Farkas H Vena GA. Diagnostic approach to HAE with normal C1-INH: consensus from HAE International. Allergy 2022;77:3175–88.

[8] Glassman F Feuersenger H Magerl M. Pharmacokinetics, pharmacodynamics, and safety of subcutaneous and intravenous garadacimab following single-dose administration in healthy Japanese and White adults. J Clin Pharmacol 2024;64:789–99. 10.1002/jcph.6162 PMC11937999 39582204

[9] Marceau F Regoli D. Bradykinin receptor ligands: therapeutic perspectives. Pharmacol Rev 2020;72:932–82. 10.1038/nrd1522 15459675

[10] Craig T Magerl MA Levy DS. Prophylactic use of an anti-activated factor XII monoclonal antibody, garadacimab, for patients with C1-esterase inhibitor-deficient hereditary angioedema: a randomised, double-blind, placebo-controlled, phase 2 trial. Lancet 2022;399:945–55. 35219377 10.1016/S0140-6736(21)02225-X

[11] Craig TJ Levy DS Reshef A. Long-term efficacy, safety, and quality-of-life data from a phase 2 randomized, open-label extension trial of garadacimab for hereditary angioedema prophylaxis. Lancet Haematol 2024;11:e436–47. 38710185 10.1016/S2352-3026(24)00081-4

[12] Zuraw BL Bernstein JA Craig T. Garadacimab in hereditary angioedema: a randomized, double-blind, placebo-controlled Phase 3 study (VANGUARD). N Engl J Med 2024;390:12–21.

[13] Lumry WR Manning ME Davis-Lorton M. Health-related quality of life and patient-reported outcomes in hereditary angioedema. Allergy Asthma Proc 2022;43:398–404.

[14] Beuschlein F Hack CE Kottke-Marchant K. FXIIa inhibitors in inflammatory and thrombotic conditions: beyond HAE. Thromb Haemost 2023;123:567–76.

